# *Oreocnide integrifolia* Flavonoids Augment Reprogramming for Islet Neogenesis and **β**-Cell Regeneration in Pancreatectomized BALB/c Mice

**DOI:** 10.1155/2012/260467

**Published:** 2012-02-27

**Authors:** Ansarullah, Bhavna Bharucha, Malati Umarani, Mitesh Dwivedi, Naresh C. Laddha, Rasheedunnisa Begum, Anandwardhan A. Hardikar, A. V. Ramachandran

**Affiliations:** ^1^Department of Zoology, Faculty of Science, The M. S. University of Baroda, Gujarat Vadodara 390002, India; ^2^Lab No. 10 Stem Cells and Diabetes Section, National Centre for Cell Sciences, Maharashtra Pune 411007, India; ^3^Department of Biochemistry, Faculty of Science, The M. S. University of Baroda, Gujarat Vadodara 390002, India; ^4^Diabetes and Pancreas Biology Group, The O'Brien Institute and The University of Melbourne, 42 Fitzroy Street, Melbourne, VIC 3065, Australia; ^5^Division of Metabolic Endocrinology, Department of Zoology, Faculty of Science, The M. S. University of Baroda, Gujarat Vadodara 390002, India

## Abstract

Agents which can either trigger proliferation of **β**-cells or induce neogenesis of **β**-cells from precursors would be of pivotal role in reversing diabetic manifestations. We examined the role of flavonoid rich fraction (FRF) of *Oreocnide integrifolia* leaves using a mice model of experimental regeneration. BALB/c mice were subjected to ~70% pancreatectomy (Px) and supplemented with FRF for 7, 14, and 21 days after pancreatectomy. Px animals displayed increased blood glucose levels and decreased insulin titres which were ameliorated by FRF supplementation. FRF-treated mice demonstrated prominent newly formed islets budding off from ducts and depicting increased BrdU incorporation. Additionally, transcripts levels of Ins1/2, Reg-3**α**/**γ**, Ngn-3, and Pdx-1 were upregulated during the initial 1 week. The present study provides evidence of a nutraceutical contributing to islet neogenesis from ductal cells as the mode of **β**-cell regeneration and a potential therapeutic for clinical trials in management of diabetic manifestations.

## 1. Introduction

Pancreatic acinar and islet components differentiate by an epithelial-mesenchymal interaction involving a foregut endodermal evagination and the splanchnic mesoderm [1]. During this process, multipotent stem cells differentiate into exocrine and endocrine phenotypes [2]. Islet endocrine cells are purported to develop from embryonic duct-like cells by a process of budding resulting in islet morphogenesis in the duct-associated mesenchyme [3], and subsequent orchestrated sequence of gene action lays down the various classes of endocrine cells in the islet. Postnatal and adult *β*-cells seem to have poor replicative capacity as they are of a terminally differentiated state, with only about 3% of cells exhibiting potential for proliferation [4].

Decreased *β*-cell number or functioning is characteristic of diabetes mellitus, whereas an actional reduction in *β*-cell number by chemical insult or infection is the cause of hypoinsulinemia and type 1 diabetes. Diet or obesity-induced type 2 diabetes results in initial islet compensation with the consequent effect of decreased *β*-cell functioning and destruction due to islet decompensation. It is clear by now that a reduction in *β*-cell number to a critically low level is correlatable with progression and outcome of diabetes [5–7]. Roughly about 80% of islet cell population is represented by *β*-cells, and the final *β*-cell mass is established during prenatal life through sequential steps of commitment to pancreatic, endocrine, and then *β*-cell progenitors followed by differentiation and proliferation of specified cells [8]. Though *β*-cell insufficiency associated with diabetes in general was rectified by islet transplantation [9], alternative approaches involved stimulation of regeneration of endogenous pancreatic *β*-cells. Three different processes targeted to meet this alternative approach include (1) proliferation of preexisting *β*-cells, (2) neogenesis from intrapancreatic progenitor stem cells, and (3) transdifferentiation from pancreatic acinar cells. Experimental induction of *β*-cell regeneration or neogenesis in diabetic individuals finds basis from the fact that, pancreas has the potential to respond to changes in islet cell mass and regenerate *β*-cells by a probable intra-pancreatic “sensing” mechanism [10, 11]. There is sufficient evidence for presence of islet stem cells or progenitors in adult pancreas located within or near pancreatic ducts that can under appropriate stimuli differentiate into endocrine cells [12–14]. Even other workers have provided evidence for presence of islet progenitor cells and the possible ability of these cells to form mature functional islets in streptozotocin diabetic mice [12, 15].

Since insulin deficiency is the prime basis of all diabetic manifestations, strategies that can trigger *β*-cell regeneration/neogenesis would be of pivotal significance in therapy and cure of diabetes. In this behest, agents which can either trigger proliferation of *β*-cells or induction of neogenesis of *β*-cells from precursors would be of great merit in reversing diabetic complications. Reports have appeared in recent times regarding inductive agents that have been shown to stimulate regeneration and replenishment of islet cells. Fernández-Alvarez et al. [16] have in this context demonstrated stimulated regeneration within pancreas of streptozotocin-treated neonatal mice by tungstate.

Plants have always been an exemplary source of drugs, and many of the currently available drugs have been derived directly or indirectly from them. A wide array of plant-derived active principles representing numerous chemical compounds has demonstrated activity consistent with their possible use in the treatment of NIDDM [17–21].

Our studies with* Oreocnide integrifolia* (OI) extract, in terms of hyperglycemia metabolic alterations, and dyslipidemia, in type 1 and type 2 diabetes models of animals, have been encouraging [22]. Hence, it was thought pertinent to evaluate the probable islet regenerative potential of flavonoid rich fraction (FRF) of *Oreocnide integrifolia *in pancreatectomized animal model. To this end, BALB/c mice have been subjected to subtotal pancreatectomy (70%) and the regenerative ability tested by administering FRF to pancreatectomized mice.

## 2. Materials and Methods

### 2.1. Plant Material

Fresh green leaves were collected during the month of October from Imphal district (Manipur) and authenticated by botanist Dr. Hemchand Singh, D.M. College of Science, Manipur University. A voucher specimen (#344) of the herbarium has been deposited at the same department for future reference.

### 2.2. Flavonoid Rich Fraction

Briefly, one hundred g of air-dried leaves were ground to fine powder and soaked in 70% ethanol for 24 h with continuous stirring. The soaked mixture was filtered using Whatmann Number 1 filter paper and the pellet discarded after centrifugation of the filtrate at 10,000 rpm at room temperature (25°C). The supernatant was concentrated in vacuo by means of a rotavapor and then dissolved in as little water as possible and washed three times with chloroform. The resultant residual layer after extraction three times with ethyl acetate and subjected to concentration *in vacuo* served as the flavonoid rich fraction (FRF).

### 2.3. HPLC Fingerprint of Flavonoidal Fraction

**Figure 1:**
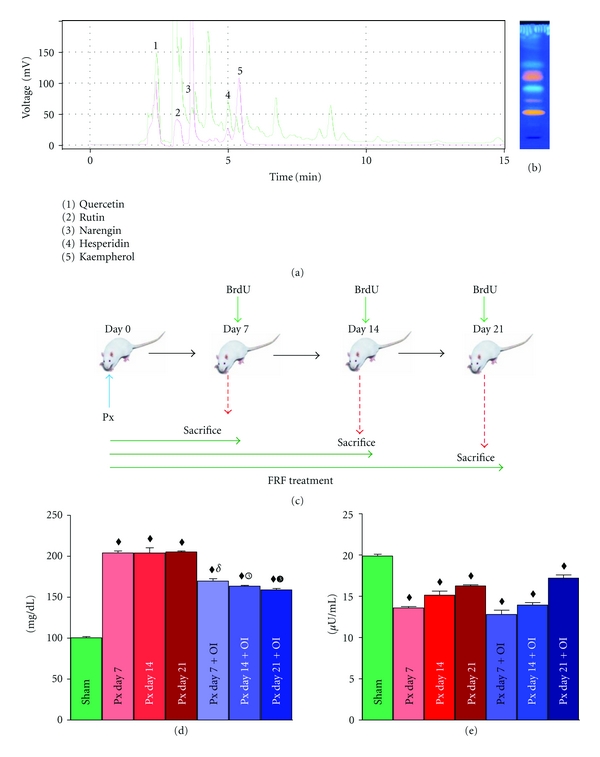
Represents (a) HPLC fingerprint of flavonoid rich fraction with marker compounds, (b) HPTLC fingerprint of Flavonoidal fraction derivatized with NP-PEG reagent, (c) schematic representation of experimental paradigm, (d and e) plasma blood glucose levels and insulin titers of Px and Px + FRF groups. All values are expressed as mean + SEM of six animals (one-way ANOVA) where, ^•^ *P* < 0.05, ^*▪*^ *P* < 0.01, ^*♦*^ *P* < 0.001: sham was compared to other experimental groups; ^*δ*^ *P* < 0.001: Px day 7 was compared Px day 7 + FRF; white numbered 3 circle *P* < 0.001: Px day 14 was compared to Px day 14 + FRF; black numbered 3 circle  *P* < 0.001: Px day 21 was compared to Px day 21 + FRF.

Chromatography was performed on Shimadzu (Shimadzu Corporation, Kyoto, Japan) chromatographic system equipped with Shimadzu LC-20AT pump and Shimadzu SPD-20AV absorbance detector. Samples were injected through a Rheodyne 7725 injector valve with fixed loop at 20 *μ*L. Data acquisition and integration was performed using Spinchrome software (Spinco Biotech, Vadodara). HPLC grade acetonitrile (ACN) and methanol (Spectrochem Ltd, Mumbai), analytical grade ammonium acetate (Merck, India), purified water from Millipore Milli-Q system were used for the experiment. For qualitative analysis, about 20 mg sample (flavonoid rich fraction) along with standards (flavonoids) were dissolved in methanol and filtered through a 0.22 *μ*m Millipore membrane filter. HPLC separation of flavonoidal rich fraction was performed at room temperature on a reverse phase C18 (250 mm × 4.6 mm i.d., 5 *μ*m particle size) column with mobile phase composed of 0.5% v/v phosphoric acid : methanol (50 : 50). The flow rate was set to 1.0 mL/min, and UV detection was carried out at 254 nm. The injection volume was 20 *μ*L (Figure 1(a)).

## 3. HPTLC Fingerprint of Flavonoidal Fraction

A Camag HPTLC system equipped with an automatic TLC sampler (ATS 4), TLC scanner 3 integrated with software (WinCATS version 1.4.2), and UV cabinet and automatic developing chamber ADC2 with humidity control facility was used for the analysis. The samples were applied using automated TLC sampler in 10 mm bands, 10 mm from the bottom, and 15 mm from the sides, with 8 mm space between the two bands. Plates were developed in software-controlled Camag automatic developing chamber ADC2 presaturated with 10 mL of developing solvent phase for 30 min at room temperature (25°C), and relative humidity was maintained (45%). The plates were developed in ethyl acetate : formic acid : glacial acetic acid : water; 100 : 11 : 11 : 26 (v/v). NP-PEG reagent was used to derivatize flavonoids (Figure 1(b)).

### 3.1. Animals and Operative Procedures

 Adult female mice of BALB/c strain around 7-8 weeks old (25–27 gm) were obtained from Cadila Pharmaceuticals, Ahmedabad, Gujarat, and acclimatized in the department of animal house for one week. The experiment was carried out according to the guidelines of the Committee for the Purpose of Control and Supervision of Experiments on Animals, India, and approved by the Animal Ethical Committee of Department of Zoology, The M.S. University of Baroda, Vadodara (Approval Number 827/ac/04/CPCSEA).

 Partial pancreatectomy (Px) was performed according to the procedures of Bonner Weir et al. [23] and Hardikar et al. [24]. Briefly, mice were anesthetized using ketamine (150 mg/kg) and xylazine (10 mg/kg) intraperitoneally. A midline abdominal incision allowed exteriorization of the splenic lobe of the pancreas (between the gastroduodenal junction and the spleen). Approximately ~70% percent pancreatectomy was performed by gently denuding the pancreatic tissue from the splenic lobe using cotton-tipped swabs soaked in 0.9% saline, leaving the mesentric pancreas intact. Incisions were closed using 5–0 catgut absorbable sutures (Ethicon), and 3 M Vetbond tissue adhesive. The skin was clipped using 9.0 mm skin staples (Leuckoclip SD, Australia), and topical ointment (Soframycin, Aventis Pharma. Ltd., Pune, India) was applied over the sutured wounds following surgery. Sham-operated animals were anesthetized, and the pancreas was gently held through the midline incision with cotton applicators for 60 seconds. The skin was sutured as in the case of pancreatectomized animals. All animals (pancreatectomized and sham operated) received an intraperitoneal injection of gentamycin (3 mg/kg body weight), ampicillin (20 mg/kg body weight) and were administered analgesics (buprenorphine HCl in normal saline 0.1 mg/kg subcutaneously).

### 3.2. Experimental Groups

The animals were divided into 7 groups, consisting of at least 6 animals, each and were sacrificed at predefined time points. Group 1: Sham operated; Group 2: Day Px 7; Group 3: Day Px 14; Group 4: Day Px 21; Group 5: Day Px 7 + FRF; Group 6: Day Px 14 + FRF; Group 7: Day Px 21 + FRF. FRF groups received 250 mg/kg body weight of flavonoidal rich fraction intraperitoneally starting from the day of surgery, while sham operated and pancreatectomized groups received 0.5% sodium carboxymethylcellulose as vehicle (Figure 1(c)). Animals were fasted overnight prior sacrifice. The experiment was carried out according to the guidelines of the Committee for the Purpose of Control and Supervision of Experiments on Animals (CPCSEA), India, and approved by the Animal Ethical Committee of Department of Zoology, The M.S. University of Baroda, Vadodara (Approval no. 827/ac/04/CPCSEA).

### 3.3. Glucose and Insulin Measurements

Plasma glucose was measured by the tail-snipping method using one-touch-glucometer (Elegance, USA). Plasma insulin was quantified according to manufacturer's protocol using mouse insulin ELISA kit (Mercodia Diagnostics, Uppsala, Sweden).

### 3.4. *In Vivo* BrdU Pulse Labelling

*In vivo *pulse-labelling with 5-bromo-2-deoxyuridine, a thymidine analogue, and subsequent immunostaining of the incorporated BrdU were carried to mark the cells that synthesized DNA during the incubation time. Six hours before sacrifice, BrdU labelling reagent (100 mg/kg body weight, Sigma Aldrich, MO) was injected intraperitoneally into Sham, Px and Px + FRF groups of animals to label proliferating cells. BrdU stock was prepared in phosphate buffered saline (PBS), (pH 7.2, 0.1 M) with 0.1 N NaOH at 20 mg/mL. After sacrifice, a portion of the pancreas from the same anatomical location, including the main pancreatic duct, was immediately fixed in 4% paraformaldehyde buffered with 0.01 mol/L sodium phosphate, pH 7.4 (PBS) overnight at 4°C, dehydrated with ethanol and embedded in paraffin wax.

### 3.5. Haemtoxylin and Eosin Staining

Pancreatic tissue sections were incubated at 60°C for 10 min, deparaffinised in xylene, and then stained with Harris haematoxylin (Sigma-Aldrich, St Louis, USA). Sections were then dehydrated using gradients of ethanol and finally in 100% ethanol before staining with Eosin Y (Hi-Media Labs, Mumbai, India). Slides were then rehydrated by downgrading them in ethanol and mounted in DPX. Images were captured using Leica DMRB binocular microscope with digital camera.

### 3.6. Immunohistochemistry and Confocal Microscopy

Five microns thick serial sections were mounted on Poly-L-lysine (Sigma Aldrich, MO, USA). For immunohistochemical detection of BrdU-incorporating nuclei, DNA was first denatured to expose the antigen by incubating the tissue sections in 1 N HCl for 45 min at 45°C. The sections were rinsed three times for 5 min each in PBS then incubated with primary antibodies to mouse monoclonal BrdU (1 : 200 Sigma Aldrich, MO, USA), Guinea pig anti insulin (1 : 200; Invitrogen, USA), Rabbit CK-19 (1 : 200, Abcam, USA), rabbit Pdx-1 polyclonal (1 : 400, Millipore) in 0.1% Triton-X solution in antibody diluent solution, for 12 h at 4°C. For Pdx-1, antigen unmasking step was performed in microwave using sodium citrate buffer. Nonspecific blocking was performed using 4% normal donkey serum and 1% BSA. Next, the labelled sections were washed with PBS (3 times at 5 min each), and secondary incubation was carried out in the dark for 2 hr at room temperature. For the secondary incubation, appropriate fluorochrome-conjugated secondary antibody Alexa-Fluor 488 and Alexa-Fluor 546 F(ab′)_2_ secondary antibodies (Molecular Probes, OR, USA) were used at 1 : 200 dilution. DAPI was used to visualize nuclei. Negative controls were run where the primary antibody incubations were omitted. After 2 hr, the tissue sections were washed in calcium magnesium containing PBS and mounted with antifade mounting medium Vectasheild (Vector Laboratories, Burlingame, CA). A laser scanning confocal microscope, model LSM 510 META (Carl Zeiss, Germany), was used with a 63X1.4NA pan Apochromat objective with optical Z sections was taken at ~0.8 microns. Magnification, laser and detector gains, and pinhole settings were set below saturation and were identical across samples.

### 3.7. Morphometry and Image Analysis

For proliferation index of BrdU-labelled cells, number of BrdU^+^ cells per islet was counted in the insulin positive or glucagon positive area. At least 10 islets per animal from nonoverlapping areas were counted, and islets of less than 100 microns were considered as small, islets while those that were more than 100 micrometer in size were considered as large islets. Acinar cell proliferation analysis was performed by randomly acquiring five DAPI/BrdU areas of acinar cells from each section. Acinar cells were counted for total BrdU^+^ and total DAPI^+^ nuclei in day 7. Individual animal results of pancreatic acinar tissue each represent 10 fields counted per animal BrdU incorporation in ducts was also counted in day 7. Ducts less than 100 microns (internal diameter) were considered small-duct while those of 100–300 microns were considered large ducts. Number of ducts giving rise to islets was counted using H&E staining and classified as large and small ducts based on internal diameter.

### 3.8. Quantitative Real-Time PCR

Pancreas was homogenized in Trizol (Invitrogen, Carlsbad, CA), and RNA was isolated as per the manufacturers' instructions, measured on ND-1000 spectrophotometer (NanoDrop Technologies, Wilmington, DE) and taken for reverse transcription and TaqMan-based probe quantitative real-time PCR. First strand cDNA synthesis was carried out using “high capacity cDNA archive kit” (Applied Biosystems, Foster City, CA). PCR was performed in 10 *μ*L total volume in 96-well plates using cDNA prepared from 100 ng of total RNA on a 7500 FAST real-time PCR cycler (Applied Biosystems, Foster City, CA). Primers and probes were Assay-on-Demand (Applied Biosystems, Foster City, CA). All qRT-PCR results are normalized to 18S (VIC-labelled) ribosomal RNA carried out in duplex reaction (with FAM-labelled target gene probes) to correct for any differences in RNA input. All PCR reactions were analyzed after 35 cycles of reaction. The ΔΔ Ct method of relative quantification was used to determine the fold change in expression. This was achieved by first normalizing the resulting threshold cycle (Ct) values of the target mRNAs to the Ct values of 18S internal control of the same sample (ΔCt = Ct_target_ − Ct_18*S*_). It was further normalized with the experimental control (ΔΔCt = ΔCt_target_ − ΔCt_control_). The fold change in expression was then obtained (2^−ΔΔCt^).

## 4. Results

### 4.1. Plasma Glucose and Insulin Levels

Pancreatectomized mice entered into a phase of significant hyperglycaemia within one week, which persisted even at three weeks. Pancreatectomized mice treated with FRF extract showed significantly lower hyperglycaemia, which showed a slow but gradual decline through one to three weeks (Figure 1(d)). Plasma insulin level which was significantly low one week after pancreatectomy showed a gradual increase through weeks two and three. Though there was no significant difference in plasma insulin titre of Px FRF mice at week one and two compared to Px, during the third week, the insulin level showed noticeable increase compared to Px animals (Figure 1(e)).

### 4.2. Histological Observations

**Figure 2:**
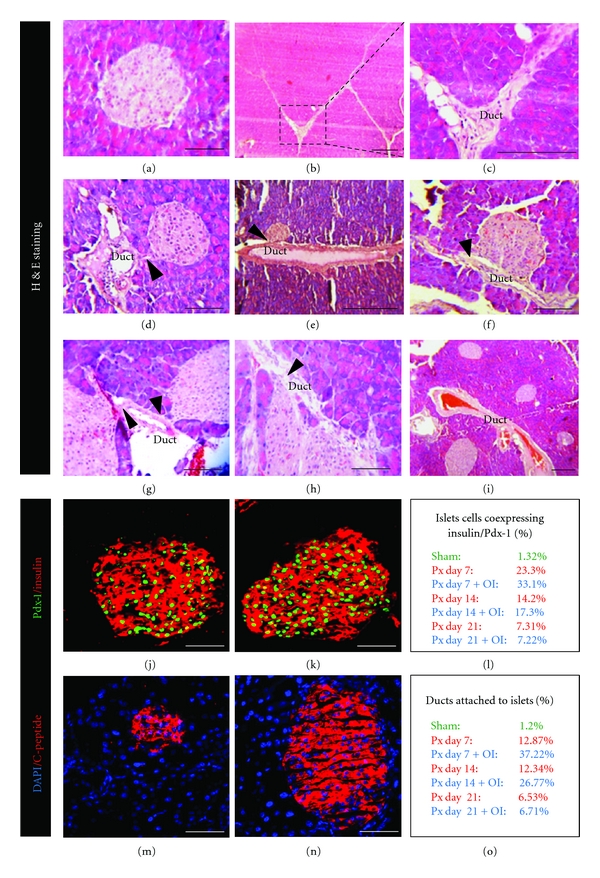
(a) Hematoxylin and eosin-stained images of pancreas from sham operated mice at day 7, (b, c) pancreatectomized mice depicting large ducts at day 14, (d)–(f) Px + FRF-treated mice at day 7 after Px showing islet neogenesis in association with ducts. Note the islet-like aggregations in contact with ducts suggesting ductal origin. Black arrows denote the junction of duct and islets, (g, h) demonstrate sections of Px + FRF supplemented mice 14 days after Px. Note the larger-sized ducts associated islets with proliferating cells in both ducts and islets and (i) sections of pancreas of Px + FRF supplemented mice 21 days after Px showing many neogenic islets, budded off from the ducts and lying within the acini. Scale bar represents 50 *μ*m. (j and k) Confocal immunostained images of Px and PX + FRF-treated mice at day 7 after Px showing Pdx-1/insulin coexpression, respectively, (l) percentage of islet cells coexpressing Pdx-1/insulin. (m and n) C-Peptide stained sections of Px and PX + FRF-treated mice at day 7 after Px, (o) percentage association of ducts to islets at different time points after Px and FRF supplementation. Scale bar represents 50 *μ*m.

Evaluation of routine HE-stained sections of pancreas revealed intact islet histoarchitecture along with acinar cells (Figure 2(a)), while bunch of endocrine cells budding off from small pancreatic ductules were observed by day 7 of Px. Duct-associated vascular channels are also visible in the neighbourhood of budding endocrine cells. By day 14 (Figures 2(b) and 2(c)), the budded off chumps of endocrine cells appear as well organized larger islets. By day 21, the newly formed islets have acquired normal looking demarcated islets that seemed to be sinking into the nearby acini. FRF-treated pancreatic remnants seem to show more prominent islet-like buds getting organized around pancreatic ducts by day 7 (Figures 2(d), 2(e), and 2(f)) with increased cell density, probably by way of proliferation of ductal precursor cells. Relatively more number of buds seems to be originating in FRF-treated pancreas. Clear association with vascular channels is also visible. By 14 day after Px (Figures 2(g), and 2(h)), FRF-treated mice seem to display prominent and relatively greater acinar organizations with prominent ductal epithelia. By day 21 (Figure 2(i)), well-organized islets between acini in the vicinity of ducts are visible. The FRF-treated pancreas demonstrated increased islet neogenesis. Overall quantitation of ducts associated with islets showed 37.22% in Px + FRF mice compared to 12.87% in Px group of animals by day 7 after Px (Figure 2(o)).

### 4.3. BrdU Incorporation in Islets, Ducts, and Acinar Cells

**Figure 3:**
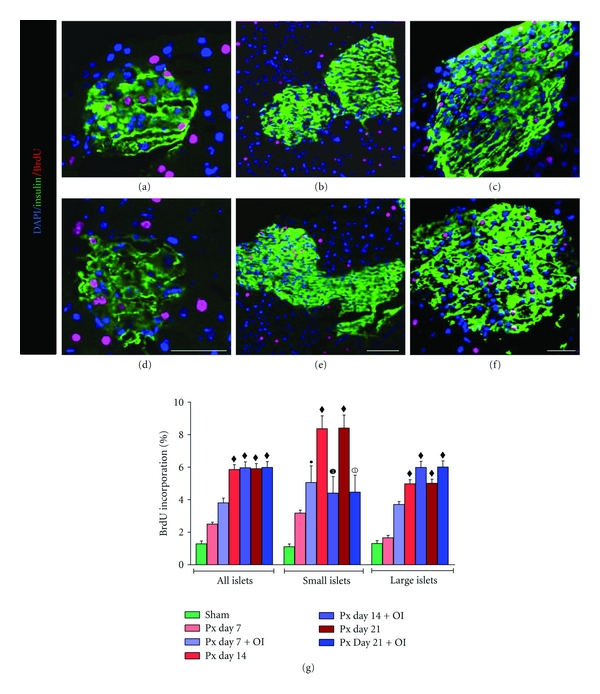
(a)–(c) Images represent insulin/BrdU immunostained confocal sections of pancreas from pancreatectomized mice at day 7, 14 and 21, respectively, and (d)–(f) Px + FRF-treated mice at day 7, 14, and 21, respectively. Scale bar represents 50 *μ*m. (g) Quantitative BrdU incorporation within islets with varying size. All values are expressed as mean + SEM of six animals (one-way ANOVA), where, ^•^ *P* < 0.05, ^*▪*^ *P* < 0.01, ^*♦*^ *P* < 0.001: sham was compared to other experimental groups; white numbered 1 circle *P* < 0.05: Px day 14 was compared to Px day 14 + FRF; black numbered 1 circle *P* < 0.05: Px day 21 was compared to Px day 21 + FRF.

There was a significant increase in BrdU incorporation by day 7 to day 21 in Px + FRF-treated mice (Figures 3(d), 3(e), and 3(f)) compared to Px group (Figures 3(a), 3(b), and 3(c)) especially in islets clusters <100 *μ*m in size. An overall quantitative evaluation of BrdU labelling in terms of percentage incorporation revealed a temporal increase across all sizes of islets (Figure 3(g)) with a significantly greater incorporation in Px + FRF pancreas.

**Figure 4:**
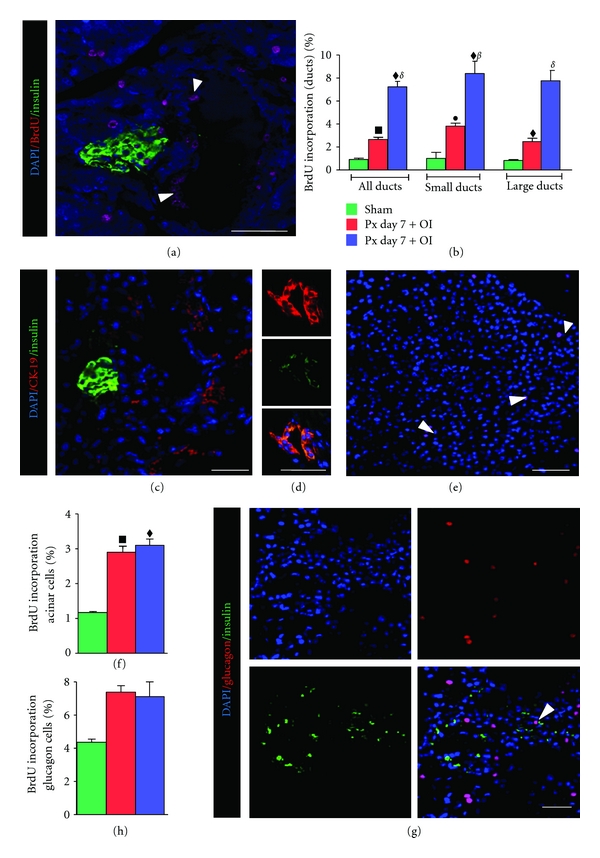
(a)–(d) Images represent BrdU incorporation in ducts, (e, f) acinar, and (g, h) glucagon cells after day 7 Px. White arrows denote BrdU-incorporated cells. Scale bar represents 50 *μ*m. All values are expressed as mean + SEM of six animals (one-way ANOVA), where, ^•^ *P* < 0.05, ^*▪*^ *P* < 0.01, ^*♦*^ *P* < 0.001: sham was compared to other experimental groups; ^*β*^ *P* < 0.01, ^*δ*^ *P* < 0.001: Px day 7 was compared to Px day 7 + FRF.

 Insulin immunoreactivity along with BrdU incorporation in ducts (Figure 4(a)) by day 7 after Px showed clearly increased BrdU labelling. A quantitative estimate of BrdU label in the ducts revealed significantly higher labelling in Px + FRF than in Px pancreas (Figures 4(a) and 4(b)). In comparison, smaller ducts seemed to show relatively higher labelling than the larger ducts (Figure 4(c)). Moreover, Px + FRF-treated mice demonstrated CK-19/insulin coexpression within ducts (Figure 4(d)).

In terms of BrdU incorporation, glucagon positive cells showed equal degree of proliferation in both Px and Px + FRF pancreas (Figures 4(g) and 4(h)). Almost same was the case even for acinar cells, though an insignificantly higher incorporation was the feature in Px + FRF pancreas (Figures 4(e) and 4(f)).

### 4.4. Immunolabelling of Insulin/Pdx-1, C-Peptide

Both Px and Px + FRF showed increment in insulin and Pdx-1 immunoreactivity seven days after Px (Figures 2(j) and 2(k)). However, Px + FRF group showed 33.1% islet cells coexpressing Pdx-1/insulin compared to 23.3% in Px animals after 7 days after Px. (Figure 2(l)). Further, C-peptide immunostaining was carried to visualize functional islets (Figures 2(m) and 2(n)).

### 4.5. Proinsulin 1 and 2 Transcripts

**Figure 5:**
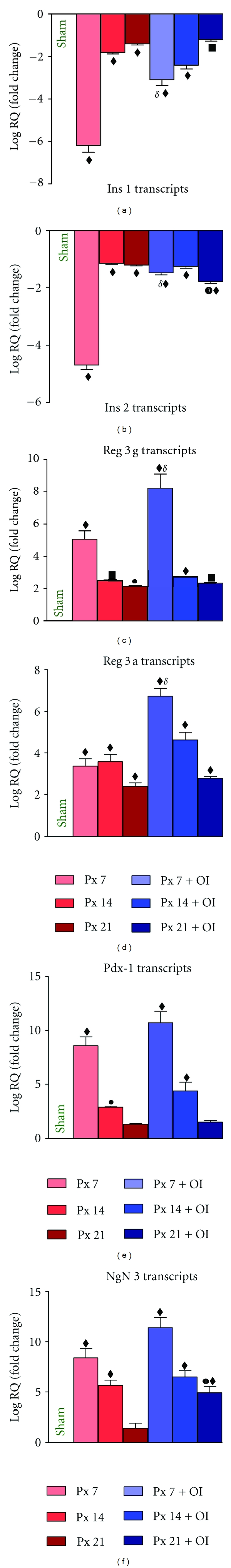
Transcript levels of (a) Proinsulin-1, (b) Proinsulin-2, (c) Reg-3 alpha, (d) Reg-3 gamma, (e) Pdx-1 and (f) NgN-3 at different time points along with FRF treatment assessed using Taqman quantitative real time pcr. Results are represented as fold change over sham operated controls. The ΔΔ Ct method of relative quantification was used to determine the fold change in expression. All values are expressed as Mean + SEM (One way ANOVA), *n* = 5-6, where; ^•^ *P* < 0.05, ^*▪*^ *P* < 0.01, ^*♦*^ *P* < 0.001: Sham was compared to other experimental groups; *δ* *P* < 0.001: Px day 7 was compared Px day 7 + FRF; black numbered 1 circle *P* < 0.05, black numbered 3 circle *P* < 0.001: Px day 21 was compared Px day 21 + FRF.

Both the proinsulin transcripts showed a significant decrement seven days after Px with a relatively lesser decrement in Px + FRF pancreas (Figures 5(a) and 5(b)). Expressions were increased gradually over 14 and 21 days after Px with significantly high expression in FRF treated pancreas.

### 4.6. Reg-3*α* and Reg-3*γ* Transcripts

Both Reg-3*α* and Reg-3*γ* transcripts were upregulated maximally at day 7 after Px with FRF-treated mice pancreas showing significantly high expression. Both the transcripts decreased gradually to reach lowest levels by day 21 after Px with the levels in Px + FRF being relatively higher at all times (Figures 5(c) and 5(d)).

### 4.7. Pdx-1 and Ngn-3 Transcripts

Upregulated expression of both transcripts were the feature at day 7 after Px with relatively greater expression in Px + FRF mice pancreas. Transcript levels of both genes decreased thereafter through day 14 to reach the lowest levels at day 21, with the transcript levels in Px + FRF pancreas being relatively higher at all time periods (Figures 5(e) and 5(f)).

## 5. Discussion

The present study on pancreatectomized (70%) BALB/c mice from day 7 to day 21 provides an evidence for a ductal contribution in islet neogenesis with specific expression of regeneration and proliferation promoting genes. Literature, in recent times, is replete with a spectrum of experimental evidences and contentions supporting differing hypotheses of islet/*β*-cell formation under a variety of natural and experimental conditions. These studies have tended to suggest widely differing mechanisms like islet/*β*-cell neogenesis from ductal precursors/stem cells [25–27] proliferation of differentiated islet *β*-cells or intraislet precursor *β*-cells [28–30], acinar and stellate cells [31, 32], or even both acinar and ductal cells [33]. Removal of 70% pancreatic mass in BALB/c strain of mouse is clearly marked by a definitive regenerative reconstitution of pancreatic mass by significant islet neogenesis from ductal cells. The histological observations provide compelling evidence for nodular budding of differentiating endocrine cells that get organized as definitive islets by 21 days after Px. Histological observations further provide convincing evidence for quantitatively greater number of neogenic islet nodes and *β*-cell density in the pancreas of mice treated with flavonoid rich fraction, suggesting the additional stimulatory role of OI extract flavonoids over and above that of the stimulatory trigger provided by the substantial decrement in pancreatic mass. Augmented rates of cell proliferation in ductal precursor cells in Px pancreas and even higher proliferation in Px + FRF are clearly indicative by the recorded higher BrdU incorporation and BrdU labelling indices. The percentage increase in BrdU incorporation seen herein provides strong evidence for the greater potential of FRF to augment ductal precursor/stem cell proliferation on subtotal pancreatectomy.

 The fact that BrdU incorporation is seen even in presumptive islets associated with ducts also denote continuing proliferation of committed islet cells (*β*-cells) even after budding off from the ducts. Apparently, islet growth and increase in cell mass could occur by proliferation of immature/young *β*-cells. Such intraislet proliferation of *β*-cells is documented even in other conditions of *β*-cell regeneration. Comparatively, proliferation within smaller islets is more intense than in larger islets. Apparently, smaller islets represent newly budded neogenic endocrine cell clusters from the ducts, and hence the tempo of proliferation initiated within the ducts due to triggering of initiation of pancreatic regeneration still persists. Though there is a slowing down of the tempo of proliferation within the larger follicles, proliferative increment of *β*-cell mass nevertheless continues. Though the BrdU incorporation studies also identify acinar cell proliferation during pancreatic restoration after surgery, their contribution to islet morphogenesis is almost nil in the present set up of pancreatic regeneration induced by subtotal pancreatectomy. However, there are some evidences for contribution of acinar cells in islet *β*-cell formation by transdifferentiation under conditions of neoplastic conversion through EGFR signaling [32], under *in vitro* conditions [34, 35].

 It is clear by now that pancreatic dysfunctioning, like in cases of pancreatectomy, pancreatitis, or pancreatic ductal obstruction, is characterized by activation of endogenous stimulators of pancreatic regeneration that may involve soluble autocrine, paracrine, and juxtacrine modulators [24, 36]. Some of the trophic factor therapies employed for *β*-cell/islet regeneration are INT, a combination of gastrin and epidermal growth factor [37, 38], glucagon-like peptide-1(GLP-1) [39], and INGAP [40, 41]. What stimulus is provided by surgical stress of pancreatectomy or, loss of a critical mass of pancreas is not clear though, it is clear from the observations that a powerful inductive regeneration signal is generated. Since a powerful environmental milieu needs to be created for bringing about islet neogenesis and pancreatic regeneration, generation of a number of initially exclusive and/or inclusive signals can be considered as a distinct possibility. It is worth contemplating on the possibility of Pdx-1 reduction as one of the possible cues for islet neogenesis in this scenario. Compelling thrust for such a consideration is provided by the fact that Pdx-1 reduction is a distinct possibility consequent to more than 70% pancreas ablation/loss as this transcription regulator is expressed in mature *β*-cells for maintenance of *β*-cell functions and prevention of glucagon production. Pdx-1 is an important transcription factor essential initially for early delineation of pancreas from the posterior foregut endodermal analogue [42] and later for maturation of endocrine lineages and *β*-cell differentiation [43]. Though ductal precursor cell proliferation is triggered by decrease in Pdx-1 and other inducer trophic factors, the ultimate effector molecules appear to be islet neogenesis-associated protein (INGAP) and/or other related group-three regeneration promoting proteins (Reg 3), which make the proliferating ductal cells to differentiate, grow into a mass, and bud off from the duct to form islet-like aggregations [35, 44]. Though a superfamily of regeneration-promoting proteins, the Reg superfamily consisting of Reg-1, Reg-2, Reg-3 isoforms, and Reg-4 and IGNAP are all reported to find expression in pancreas during *β*-cell regeneration [32, 44], a careful consideration of these reports reveals the fact that the expressions of these genes/proteins are related to the type of pancreatic insult and further that Reg-1, Reg-2, and Reg-3*β* expressions are more characteristic of intraislet *β*-cell proliferation and regeneration, while Reg-1, Reg-3*α*, and Reg-3*γ* expressions are more under conditions of islet neogenesis from nonislet sources. In this context, the present study clearly shows higher expression levels of Reg-3*α* and Reg-3*γ* transcripts after pancreatectomy. Though both the genes are upregulated, Reg-3*γ* upregulation seems to be relatively of a greater degree than Reg-3*α* and that maximal expression is recorded on day 7 after Px with gradual decline through 14 and 21 days after Px. The degree of upregulation of both the genes is significantly greater in Px + FRF mice than in Px mice. Apparently, the flavonoid rich fraction exerts an additive effect on transcriptional activation. The observed greater degree of ductal cell proliferation as assessed by BrdU incorporation and the greater number of duct associated nodular buds and the greater number of islets in Px mice exposed to FRF in comparison to nonexposed mice agree well with the highly augmented Reg-3*α* and Reg-3*γ* expressions. Islet neogenesis would, however, require specific expression of endocrine precursor commitment gene to guide the neogenic precursor cells to differentiate into islet specific endocrine cells. Studies on embryonic development of pancreas have identified neurogenin-3 (Ngn-3), a bHLH transcription factor, as the gene that commits the Hnf-1*β* ^+^ transition duct cells to endocrine progenitors [38].

In the present study, Ngn-3 mRNA expression is significantly upregulated by 8-fold at day 7 after Px, which gets reduced to 6-fold by day 14 and to a mere 2-fold by day 21. In contrast, FRF-treated Px mice show nearly 12-fold upregulation at day 7 after Px, and the upregulated expression persists at 5-6-fold high even at 14 and 21 days after Px. This would suggest islet neogenesis after Px in BALB/c mice exposed to FRF, and this find-excellent correlation with the observed duct-associated islet neogenesis following Px and the relatively greater number and size of differentiating islets in FRF-treated Px mice. Not only is Ngn-3 expression noticed at the time of endocrine specification during pancreatic morphogenesis [25, 45], but, also during islet neogenesis during pancreatic regeneration in the adult [46].

 The importance of Ngn-3 in the specification of precursor cells for the various endocrine lineages in pancreatic islets is fully established by the reported inhibition of islet neogenesis by miRNA-induced Ngn-3 gene silencing [47, 48]. It has also become clear from some recent studies that models of diabetic animals in which intraislet precursor or adult *β*-cell proliferation plays a primary role in *β*-cell regeneration, Ngn-3 expression is not important as Ngn-3 inhibition was of no consequence and normal *β*-cell regeneration occurred [29, 47]. It is also of interest to note that a higher glucose level facilitates pancreatic endocrine cell development by potentiating the expression of Neuro-D, a downstream target of Ngn-3 [49]. Presumably, as the hyperglycemia characteristic of Px as in the present case can favour islet endocrine cell development by complimenting the function of Ngn-3. The higher and larger duration persistence of Ngn-3 expression in FRF-treated mice as well correlatable with the noticed larger number and size of neogenic FRF-treated pancreas as substantiated by the documented greater immunoreactivity for insulin.

 The greater insulin immunoreactivity in the islets of FRF-exposed mouse pancreas again finds correlation with the recorded relatively lesser inhibition of Ins-1 and 2 proinsulin transcripts. The greater degree of inhibition of insulin biogenesis seen in Px mice is effectively minimized by FRF exposure. Apparently, treatment with FRF extract has significant favourable influence on insulin synthesis as seen by the relatively higher plasma insulin titres in FRF-treated mice. This increasing proinsulin mRNA expression and recovering insulin levels may suggest greater number of *β*-cells as also evidenced by the greater immunoreactivity for insulin in FRF-exposed mice, which may bear some relevance in the context of recently reported new function of Pdx-1 [50]. According to their finding, the C terminus of Pdx-1 has a novel function of forming a molecular complex with Hnf6 and inducing the expression of Ngn-3 and it also indirectly regulates ngn-3 expression by controlling the regulatory network of Sox-9, Foxa2, Hnf6, and Hnf1b. From this ability of Pdx-1 to upregulate the expression of Ngn-3 and also its known role in proliferating and differentiated *β*-cells, it is easily inferable that the presently observed greater number of islets and *β*-cells with the FRF supplementation is relatable with the greater Pdx-1 expression as substantiated by the immunocytochemical and qPCR results. This inference finds further substantiation by the herein recorded minimal glucagon positive cells and similar number of *α*-cells proliferation as detected by BrdU incorporation in both Px and Px + FRF mice.

Overall, the present findings indicate islet neogenesis as the mode of *β*-cell regeneration in pancreatectomized BALB/C mice and provide evidence for flavonoidal rich fraction to have enhancing influence on islet neogenesis and greater *β*-cell regeneration by as yet unknown influence on the genetic network controlling islet neogenesis and *β*-cell differentiation. The reports of Ogata et al. [51] and Kojima and Umezawa [52] of the ability of caryophylline a *Vinca* alkaloid in inducing differentiation of pancreatic endocrine precursor cells and of Sidhu et al. [53] of Rosiglitazone to promote *β*-cell regeneration from stellate cells are relevant in the present context. It is also clear from the present findings that, ductal cells can serve as progenitors for islet neogenesis in response to appropriate triggering stimulation(s) generated by the stress of substantial pancreatic loss, by reverting to a plastic phenotype state similar to embryonic progenitors, as has been suggested by a working hypothesis extended by Bonner-Weir and Sharma [54]. This also overrules the myth of resistance to nuclear reprogramming of fully differentiated cells and, as such, reprogramming of pancreatic exocrine cells has been adequately documented [55].

 More focused and reshaped experimental approaches need to be undertaken to fully fathom the cell types involved in reprogramming and the specific signals generated under various types of pancreatic stress and the underlying genome organization that need to be attend for reprogramming, as also opined by Bukys and Jensen [56].
